# Innovative molecular intervention and precision therapy for atherosclerosis

**DOI:** 10.3389/fcvm.2025.1652933

**Published:** 2025-10-15

**Authors:** Zhijie Wang, Wei Peng, Linsheng Huang, Yan Zhang, Jie Yang, Xiaolin Chen, Xiang Liu, Feifeng Li, Qiong Zhang

**Affiliations:** ^1^ Hubei Key Laboratory of Wudang Local Chinese Medicine Research, Hubei University of Medicine, Shiyan, China; ^2^ Department of Drug Quality Inspection, School of Pharmaceutical Sciences, Hubei University of Medicine, Shiyan, China; ^3^ Department of Preventive Medicine, School of Public Health, Hubei University of Medicine, Shiyan, China; ^4^ Department of Hepatobiliary Pancreatic Surgery, Taihe Hospital, Hubei University of Medicine, Shiyan, China; ^5^ Health Management Center, Shiyan Renmin Hospital, Hubei University of Medicine, Shiyan, China

**Keywords:** atherosclerosis, PCSK9, nanoparticles, statin, MSCs

## Abstract

Atherosclerosis is a chronic vascular disorder characterized by the pathological accumulation of lipids, inflammatory cells, and extracellular matrix within arterial walls. With the escalating global incidence of atherosclerosis, the development of more effective therapeutic interventions has emerged as a critical priority in biomedical research. Conventional treatment modalities, encompassing pharmacological agents and, endovascular interventions, have demonstrated partial efficacy in disease management. However, their clinical utility remains constrained by suboptimal therapeutic outcomes, treatment-related adverse effects, and instances of therapeutic failure. In response to these limitations, contemporary research has shifted focus toward novel therapeutic strategies targeting molecular pathways and immunomodulatory mechanisms, aiming to achieve enhanced precision and efficacy. This review synthesizes recent innovations in atherosclerosis therapeutics. Notable advancements include PCSK9 inhibitors and next-generation lipid-modulating agents, which have shown significant promise in clinical trials by achieving substantial reductions in atherogenic lipoprotein levels. Gene-editing technologies, particularly CRISPR-based approaches, exhibit potential for halting disease progression through targeted modulation of pro-atherogenic genes. Furthermore, emerging insights into the regulatory role of microRNAs in atherosclerotic plaque formation and instability have positioned miRNA-based therapeutics as a compelling frontier in precision medicine for cardiovascular diseases.

## 1 Introduction

Atherosclerosis is a chronic cardiovascular disease characterized by the convergence of endothelial injury, lipid deposition, and chronic inflammatory response, manifesting as multifocal changes that predominantly affect large and medium-sized arteries (1). The World Health Organization (WHO) reports that atherosclerosis contributes to approximately 1.7 million annual fatalities globally, representing nearly one-third of all-cause mortality worldwide (2). As a leading contributor to cardiovascular morbidity and mortality, atherosclerosis has emerged as a paradigmatic disease entity within the spectrum of cardiovascular pathologies (3).

The hallmark pathological manifestations of atherosclerosis encompass lipid deposition within the arterial intima, focal fibrotic proliferation, plaque formation, vascular wall stiffening, and luminal stenosis, collectively resulting in end-organ ischemic injury (4, 5). These pathophysiological processes are mediated through dynamic interactions among low-density lipoprotein (LDL), oxidized LDL (ox-LDL), endothelial cells, vascular smooth muscle cells (VSMCs), and associated molecular mediators.

LDL and its oxidized derivatives constitute principal drivers of atherosclerotic progression. Under pathological conditions, ApoB-containing lipoproteins infiltrate compromised vascular endothelium into the subendothelial space, where reactive oxygen species (ROS) mediate their oxidative modification (6, 7). This transformation generates ox-LDL, which accumulates within the arterial wall and initiates monocyte recruitment to the intimal layer. These monocytes subsequently differentiate into macrophages, perpetuating inflammatory cascades (8). Crucially, ox-LDL exhibits high affinity for macrophage scavenger receptors (e.g., CD36, LOX-1), enabling receptor-mediated internalization (9). Progressive phagocytosis of ox-LDL drives macrophage foam cell transformation, thereby facilitating necrotic core formation and atherosclerotic plaque expansion (10, 11).

Endothelial cells maintain vascular homeostasis through dual regulatory functions: (i) secretion of anti-angiogenic factors (e.g., nitric oxide) to suppress cellular proliferation, and (ii) production of vasoconstrictive mediators (e.g., endothelin-1) to modulate vascular tone (12–14). Concurrently, VSMCs and their synthesized collagen-rich extracellular matrix confer structural stability to advanced plaques, mitigating risks of plaque rupture and thrombotic complications (15). These mechanistic insights have directly informed current therapeutic paradigms targeting vascular remodeling.

Statins, as competitive inhibitors of 3-hydroxy-3-methylglutaryl-coenzyme. It is a (HMG-CoA) reductase, remain the cornerstone of pharmacological management for atherosclerosis in clinical practice. While the emergence of monoclonal antibody-based PCSK9 inhibitors has introduced a paradigm-shifting therapeutic strategy (16), contemporary pharmacological interventions continue to face significant clinical constraints.

Statin therapy is frequently complicated by dose-dependent musculoskeletal toxicity, encompassing a spectrum from mild myalgia (60%–70% of cases) to life-threatening rhabdomyolysis (<0.1% incidence). Severe manifestations may precipitate acute kidney injury, disseminated intravascular coagulation, and mortality, with statin discontinuation representing the sole definitive management approach for statin-associated muscle symptoms (SAMS) (17). Although PCSK9 inhibitors demonstrate favorable safety profiles in clinical trials, their therapeutic application is associated with transient adverse effects including nasopharyngitis (10%–15%), injection-site reactions (5%–7%), and upper respiratory infections (18–20). Furthermore, the cost of PCSK9 inhibitors remains prohibitively high. Therefore, the aforementioned deficiencies have largely restricted its wide application in clinical practice. At present, the comprehensive analysis systematically evaluates of intervention and therapy for atherosclerosis: (1) Optimization protocols for conventional pharmacotherapies; (2) Structural refinement strategies for next-generation inhibitors; (3) Emerging therapeutic modalities currently under preclinical/clinical investigation. Therefore, through this tripartite evaluation, we aim to delineate actionable strategies for overcoming current therapeutic limitations and inform future translational research directions in atherosclerosis management.

## 2 Therapeutic challenges of traditional therapies

Atherosclerosis is characterized by the formation of atherosclerotic plaques. Targeting this pathologically character, current clinical management primarily involves lifestyle modifications and pharmacological interventions, including statins, proprotein convertase subtilisin/kexin type 9 (PCSK9) inhibitors, anti-inflammatory agents, and antiplatelet medications.

As a commonly used traditional medicine for atherosclerosis, statins demonstrates therapeutic efficacy through significant reduction of serum low-density lipoprotein cholesterol (LDL-C) concentrations, thereby attenuating plaque development (21). However, current pharmacotherapeutic approaches present several clinical limitations: restricted cellular permeability, suboptimal aqueous solubility, and diminished bioavailability significantly compromise treatment outcomes (22). Furthermore, high-dose statin regimens are associated with adverse effects including drug intolerance, fatigue manifestations, and statin-associated muscle symptoms (SAMS), while alternative non-statin therapies frequently prove inadequate in halting disease progression (23).

These therapeutic challenges underscore the critical need for developing novel strategies to enhance statin pharmacokinetic profiles and optimize therapeutic outcomes in atherosclerotic management.

## 3 Application of nanotechnology in the optimization of traditional interventions

### 3.1 The optimization of traditional statin drugs by nanotechnology

**Figure 1:**
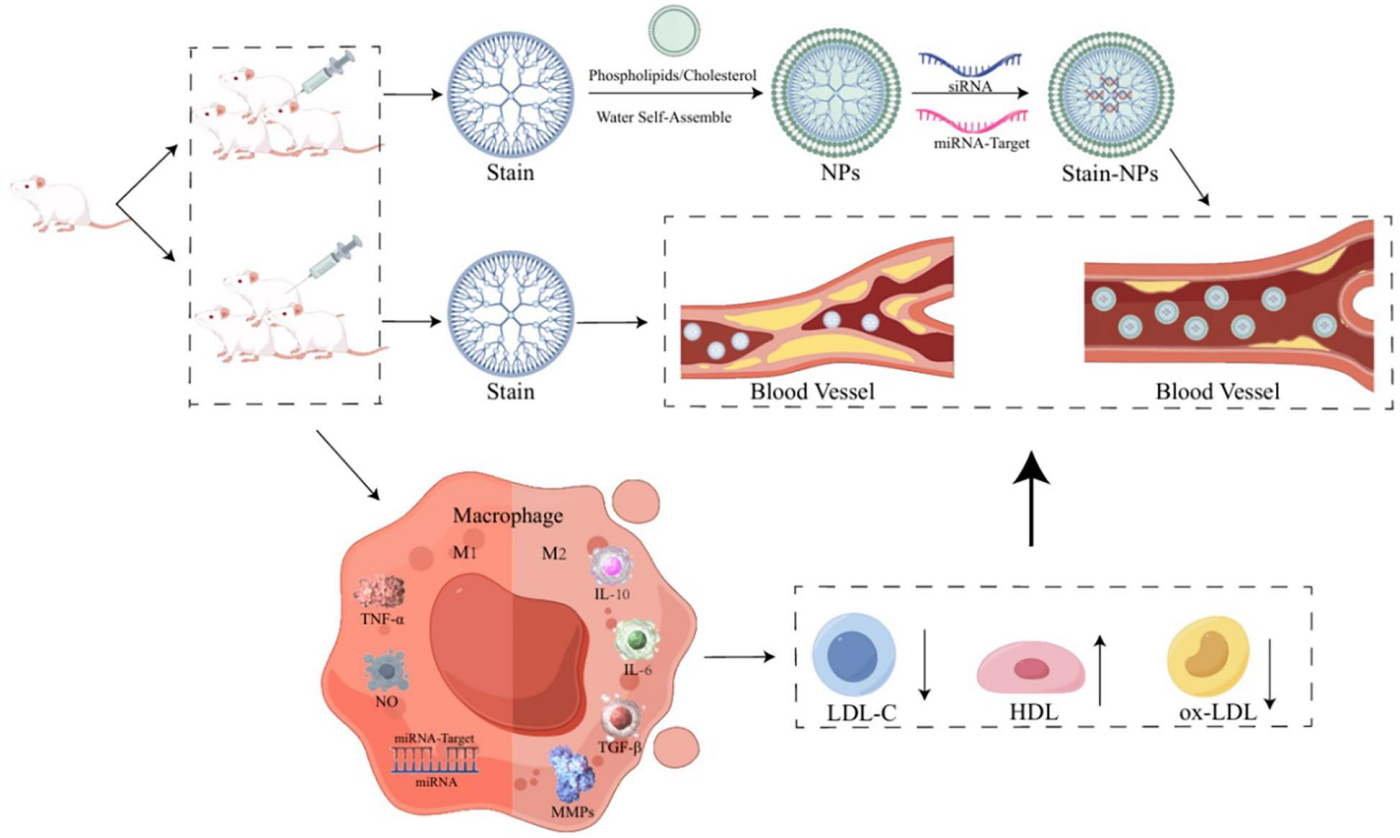
The anti-atherosclerotic performance of statins, specialized nanocarrier systems engineered for atherosclerotic microenvironments. Created using Figdraw.

The progressive evolution of nanotechnology has established engineered nanoparticles as a promising platform in cardiovascular therapeutics, leveraging their enhanced delivery efficiency, precise target selectivity, and reduced off-target effects (24). To augment the anti-atherosclerotic performance of statins, specialized nanocarrier systems engineered for atherosclerotic microenvironments have demonstrated superior therapeutic outcomes compared to conventional free drug formulations (25, 26) (Figure 1).

Building on these developments, researchers pioneered a co-delivery system integrating statins with nucleic acid therapeutics, and revealed that anti-miR-33 exhibits dual functionality as both an atheroprotective nucleic acid and a modulator of macrophage phenotypic polarization (27, 28). Through covalent conjugation of hydrophobic atorvastatin with synthetic tri-glucosyl-methyl chitosan (TMC) via amide and ester linkages, the team achieved spontaneous self-assembly of cationic nanoparticles (GTANPs) in aqueous media. Subsequent electrostatic complexation enabled efficient encapsulation of anionic nucleic acid payloads (siBaf60a and anti-miR-33 pDNA), yielding GTANPs/siBaf60a and GTANPs/pAnti-miR-33 nanocomposites (29).

This innovative methodology provides valuable mechanistic insights for advancing statin-based therapeutic strategies in atherosclerosis through nanotechnology-enabled drug optimization.

### 3.2 Nanotechnology and atherosclerosis imaging diagnosis

Molecular imaging technology enables high spatiotemporal resolution visualization of rupture-prone or erosion-susceptible atherosclerotic plaques, serving as a critical tool for both disease discovery and diagnostic evaluation (30). Beyond their established delivery efficiency, engineered nanoparticles exhibit nanoscale dimensions and enhanced tissue penetrability (31). These intrinsic properties empower nanoparticle-modified therapeutics to exploit the unique vascular permeability of atherosclerotic lesions, facilitating passive or ligand-directed active transport across endothelial barriers for targeted accumulation at pathological sites (31). Integration of these platforms synergistically enhances diagnostic precision in atherosclerosis management.

Current clinical imaging modalities for atherosclerosis primarily encompass two paradigms: structural imaging (e.g., MRI, CT) and functional imaging (e.g., PET, SPECT) utilizing radiotracer-based techniques. However, these approaches exhibit persistent limitations in differentiating vulnerable plaques from stable lesions, particularly in early-stage disease (32–34). Emerging strategies focusing on molecular engineering of contrast agents and enhancing their plaque-specific accumulation demonstrate potential for achieving superior diagnostic specificity.

Iron oxide nanoparticles (IONPs), characterized by their superparamagnetic properties, align their magnetic domains under external fields and serve as potent MRI contrast enhancers. Recent advancements confirm that surface functionalization of IONPs with inorganic coatings significantly enhances biocompatibility profiles while maintaining imaging efficacy (35). Such optimized nanocomposites exhibit exceptional performance as theranostic agents when integrated with MRI for atherosclerotic plaque detection. Notably, ferumoxytol, currently the sole FDA-approved nanoparticle for clinical imaging applications, has demonstrated remarkable translational potential in this domain (36).

## 4 Therapeutic integration and future perspectives of PCSK9 inhibitors in atherosclerosis management

Since the beginning of the 21st century, scientific research on atherosclerosis treatment has faced persistent challenges in developing novel therapeutic interventions that combine enhanced efficacy with improved safety profiles and cost-effectiveness (37, 38) (Table 1). Notably, the pandemic era has accelerated interest in nucleic acid-based therapies, with emerging research focusing on diverse non-coding RNA species, including microRNAs, lncRNAs, circular RNAs, si-RNAs, and tRNA-derived fragments, as promising therapeutic targets (39).

**Table 1:** Recent completed clinical trials with nucleic acid-based therapeutics.

Drug	Trial phase	Molecular target	Outcome metrics	Status
Evolocumab	Phase IV	PCSK9	Reduce low-density lipoprotein cholesterol levels to a median of 30 mg/dl (0.78 mmol/L) and lower the risk of cardiovascular events.	Completed
Rosuvastatin	Phase IV	HMG-CoA	–	Completed
Semantine	Phase III	NMDA	–	On going
Alirocumab	Phase I	PCSK9	There was a significant decrease in LDL-C and other related indices, and fewer adverse major events occurred.	Completed

### 4.1 Gene structure and functional mechanisms of PCSK9

#### 4.1.1 Gene structure of PCSK9

As a member of the proprotein convertase family, PCSK9 plays a pivotal role in protein hydrolysis activation, post-translational modification, and regulation of secreted protein degradation (40). Its structural architecture comprises three distinct domains: an N-terminal signal peptide (SP, residues 1–30), a propeptide domain (PD, residues 31–152), and a catalytic domain (CD, residues 153–426) (41).

The propeptide domain facilitates PCSK9 synthesis and secretion through autocleavage-mediated maturation, while maintaining post-secretion autoinhibition via non-covalent binding to the catalytic domain. Functionally, the catalytic domain features a conserved serine protease fold housing the catalytic triad (His226, Asp374, Ser386), which is essential for low-density lipoprotein receptor (LDL-R) binding (42).

#### 4.1.2 Low-density lipoprotein receptor (LDL-R) biology and pathogenic significance

The LDL-R, a transmembrane glycoprotein, is critically involved in lipoprotein metabolism, with its genetic mutations recognized as the principal etiology of familial hypercholesterolemia (FH) (43). Structurally, it consists of an extracellular ligand-binding domain, an epidermal growth factor precursor homology domain, and an O-linked glycosylation region proximal to the transmembrane helix.

The transcriptional regulation of the LDL-R gene relies on two TATA-like sequences and three conserved 16-bp direct repeats within its promoter region (44). Mechanistically, LDL-R mediates circulatory lipoprotein clearance through clathrin-coated pit endocytosis. At physiological pH, its extracellular domain adopts an extended conformation to capture apolipoprotein B-containing lipoproteins (45).

#### 4.1.3 PCSK9-LDL-R interaction dynamics on atherosclerosis management

Predominantly expressed in hepatic, intestinal, renal, and neural tissues (46), PCSK9 exerts its primary lipid-modulating effects by regulating LDL-R expression on hepatocyte membranes (47). Upon secretion, circulating PCSK9 engages LDL-R at the hepatocyte surface through pH-dependent interactions (48). Under neutral plasma membrane conditions, the catalytic domain of PCSK9 binds the EGF-A domain of LDL-R with moderate affinity (Kd = 170–750 nM), forming a 1:1 stoichiometric complex (49, 50).

Following internalization, acidic endosomal conditions enhance binding avidity by 150-fold through electrostatic interactions between the positively charged C-terminal histidine-rich domain (CHRD) of PCSK9 and the negatively charged LDL-R ligand-binding domain (51). This pH-driven conformational shift disrupts receptor recycling, diverting the PCSK9-LDL-R complex to lysosomal degradation (40, 52).

**Figure 2:**
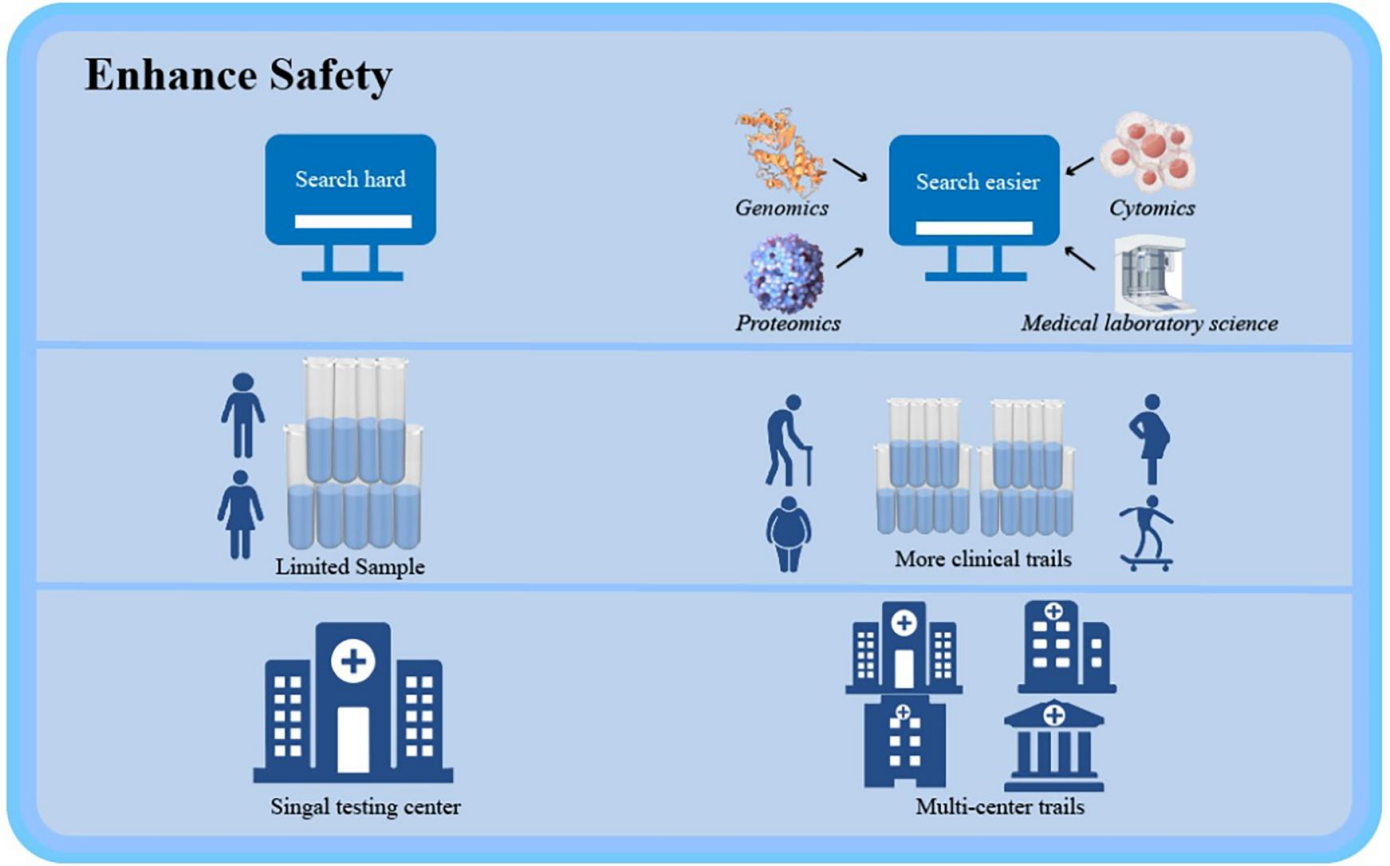
Recent advances in PCSK9 inhibitor development, particularly through integration with emerging biotechnologies, have substantially expanded therapeutic strategies for atherosclerosis management. Created using Figdraw.

Recent advances in PCSK9 inhibitor development, particularly through integration with emerging biotechnologies, have substantially expanded therapeutic strategies for atherosclerosis management (Figure 2).

#### 4.1.4 Nucleic acid drugs and gene editing drugs

Inclisiran, a first-in-class siRNA-based inhibitor, targets PCSK9 mRNA for degradation, thereby mimicking natural PCSK9 loss-of-function (LOF) and reducing PCSK9 levels (53). Approved by the FDA in December 2021, inclisiran's impact on major adverse cardiovascular events is currently being evaluated in the ORION-9, ORION-10, and ORION-11 clinical trials involving individuals with established atherosclerotic cardiovascular disease (ASCVD), with primary completion anticipated in July 2026. Meanwhile, Verve-101, the first PCSK9-targeted gene-editing drug developed by Verve Therapeutics, is in Phase I clinical trials. In the FOURIER trial, over three-quarters of enrolled patients had a history of myocardial infarction (with the indicator event occurring a median of 3.4 years prior), 19% had a history of non-hemorrhagic stroke, and 13% had peripheral artery disease (PAD). In contrast, the ODYSSEY OUTCOMES trial targeted a more acute population, enrolling patients who had recently experienced acute coronary syndrome. Despite this difference in patient acuity, the average baseline LDL-C levels were similar between the two trials: 92 mg/dl in FOURIER and 87 mg/dl in ODYSSEY OUTCOMES (54–56). This single-course treatment permanently silences the PCSK9 gene in the liver using a CRISPR-Cas9-derived editing tool that introduces A-to-G base edits at specific PCSK9 loci to disable gene function (57).

### 4.2 mRNA-based therapeutic strategies targeting PCSK9

As an emerging therapeutic modality, mRNA technology has demonstrated revolutionary potential across diverse disease domains (58). The rapid clinical translation of highly efficacious mRNA-based COVID-19 vaccines during the pandemic era has particularly highlighted its therapeutic versatility.

Of particular interest in atherosclerosis management is interleukin-10 (IL-10), a potent immunomodulator predominantly secreted by macrophages. This cytokine exerts critical regulatory effects on inflammatory responses and promotes tissue repair within atherosclerotic plaques (59, 60). Capitalizing on these properties, predecessors pioneered a macrophage-targeted nanoparticle delivery system for anti-inflammatory mRNA therapeutics. Their innovative approach utilizes pH-responsive, charge-switching polymeric nanoparticles capable of precise delivery of IL-10-encoding mRNA to plaque-associated macrophages.

Through comprehensive evaluation in a HFD model, this platform addressed two key pharmacological challenges: (1) overcoming the inherent instability and rapid systemic clearance of naked mRNA, and (2) achieving sustained therapeutic effects within the complex plaque microenvironment. These findings hold dual significance—they not only establish a robust methodology for mRNA-based modulation of local inflammatory processes but also provide a strategic framework for combining anti-inflammatory therapies with conventional LDL-C-lowering interventions (61).

### 4.3 Efficacy and safety considerations of optimizing long-term outcomes of PCSK9 inhibitors

The development of extended-action PCSK9 inhibitor formulations aims to improve therapeutic adherence by minimizing dosing frequency. Clinical surveillance data reveal characteristic adverse event profiles, with musculoskeletal pain (27.2%), nasopharyngitis (9.3%), transaminase elevation (6%), and influenza-like symptoms (7.5%) representing the most frequently reported complications (62–64). These findings underscore the imperative for optimizing both pharmacological durability and safety parameters in PCSK9-targeted therapies.

#### 4.3.1 Precision medicine approaches

Advancements in mechanistic understanding enable tailored therapeutic strategies to bridge guideline-practice disparities. Integrative multi-omics platforms, spanning genomics, transcriptomics, proteomics, and metabolomics, facilitate comprehensive biomarker discovery and systems-level analysis of drug response heterogeneity (65). Such approaches empower: (1) Identification of phenotype-specific therapeutic targets; (2) Prediction of protein-drug interactions and off-target effects; (3) Stratification of patients for personalized dosing regimens (66–68).

#### 4.3.2 Robust clinical validation frameworks

**Figure 3:**
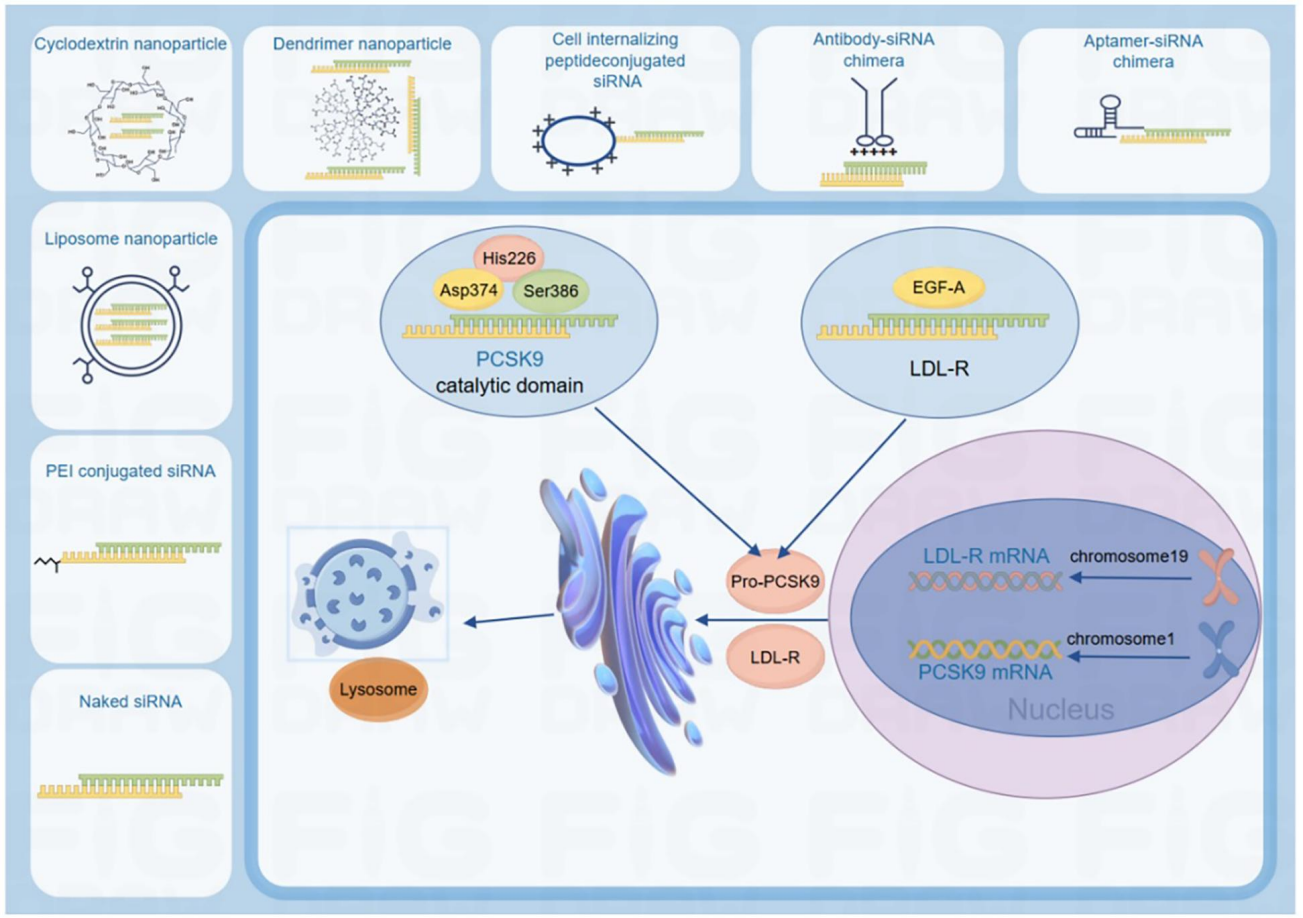
The development of molecular biology and the rigor of epidemiology provide sustainable development for the safety and long-term efficacy of PCSK9 inhibitors. Created using Figdraw.

Large-scale multicenter trials are critical for validating therapeutic outcomes across diverse populations. Key implementation strategies include: (1) Population diversification: Expanding enrollment to understudied cohorts (geriatric, gestational, high-comorbidity patients) to characterize long-term safety profiles; (2) Geographic generalizability: Incorporating multi-regional clinical practice data to ensure therapeutic consistency across ethnicities and healthcare systems. This dual focus on molecular precision and epidemiological rigor establishes a sustainable paradigm for next-generation PCSK9 inhibitor development (Figure 3).

#### 4.3.3 Clinical promise for lipid management of PCSK9 inhibitor MK-0616

The current clinical strategy encompasses FDA-approved monoclonal antibodies (evolocumab, alirocumab, bococizumab) and liver-targeted PCSK9 synthesis inhibitors (69). Among emerging therapies, MK-0616, a potent oral macrocyclic peptide PCSK9 inhibitor, demonstrates dual efficacy in reducing LDL-cholesterol, non-HDL-cholesterol, apoB, and Lp(a), while offering potential advantages over injectable alternatives through simplified dosing, enhanced patient adherence, and cost efficiency (70). Preclinically, MK-0616 exhibited high PCSK9-binding affinity (K_i_ = 5 pM), favorable safety, and sufficient oral bioavailability to support clinical translation. Phase 1 trials in healthy adults revealed that single oral doses achieved >93% geometric mean reduction (95% CI: 84–103) in free plasma PCSK9; notably, statin-treated participants receiving 20 mg MK-0616 daily for 14 days showed a maximal 61% geometric mean reduction (95% CI: 43–85) in LDL-cholesterol from baseline (71).

### 4.4 Pharmacological research on trodusquemine

Vascular smooth muscle cells (VSMCs) have been extensively studied as the primary cellular component implicated in the vulnerability of carotid atherosclerotic plaques (72). The platelet-derived growth factor receptor (PDGFR) family comprises two principal isoforms, PDGFR-α and PDGFR-β, with studies indicating that both PDGF ligands and their corresponding receptors demonstrate significantly higher expression levels in atherosclerotic vessels compared to normal controls (73–75).

#### 4.4.1 PTP1B mediates dual pathogenic roles in vascular pathophysiology

Protein tyrosine phosphatase 1B (PTP1B), a key regulatory enzyme in tyrosine phosphorylation processes, has been shown to critically modulate PDGF receptor signaling pathways. Mechanistic studies reveal that PTP1B mediates PDGF/PDGFR signal regulation in VSMCs through endocytic processing, thereby suppressing PDGF-induced hyperactivation of VSMC biological functions (76–78).

The elevated systemic PTP1B activity promotes macrophage uptake of oxidized cholesterol through scavenger receptors, facilitating their transformation into lipid-laden foam cells. This cellular transformation accelerates arterial lipid accumulation and potentiates pro-inflammatory responses within vascular tissues.

#### 4.4.2 PTP1B targeting from molecular inhibition to clinical atherosclerosis therapy

Experimental investigations using animal models, researchers demonstrated that PTP1B upregulation induces cellular apoptosis and suppresses smooth muscle cell migratory capacity in carotid arteries. Notably, KY266-treated mice (a selective PTP1B inhibitor) exhibited significant reductions in pathology-associated protein expression levels, correlating with attenuated atherosclerotic progression. These findings collectively indicate PTP1B's critical regulatory role in atherogenesis and its potential as a molecular target for therapeutic intervention (79, 80).

Structural analyses reveal that trodusquemine binds specifically to the primary binding site formed by α-helices 7 and 9 of PTP1B. This interaction triggers structural reorganization of α-helices 7, 3, and 6, generating a secondary pocket with partial overlap to the exosite. The resultant allosteric inhibition stabilizes the WPD loop in an open conformation, effectively locking PTP1B in an inactive state through dynamic domain rearrangements (80).

Recent translational research demonstrated trodusquemine's therapeutic efficacy. The compound is systematically evaluated using peripheral blood leukocytes from 30 atherosclerotic patients with confirmed coronary artery disease and 30 age-matched healthy controls, showing consistent pharmacological activity across disease states.

#### 4.4.3 Phase status or translational challenges of PTP1B

In current research, numerous novel PTP1B inhibitors have emerged, including compounds such as BDB [3-bromo-4,5-bis(2,3-dibromo-4,5-dihydroxybenzyl)-1], derivatives of 2-(naphthalen-2-yl)-1,2,5-thiadiazolidin-3-1,1-dioxide, MSI-1436 analogues like PMM-1001, and 5-(naphthalen-2-yl)-1,2,5-thiadiazolidin-3-one. However, despite their potential as therapeutic agents, these inhibitors remain at the experimental stage, with no clinical trial data currently available (81–83).

However, a major limitation of PTP1B is its lack of specificity and cellular permeability, posing significant challenges to clinical translation (84). Most inhibitors target the catalytic active site, resulting in non-specific inhibition across all protein tyrosine phosphatases (PTPs). Consequently, developing novel strategies to overcome these limitations, such as designing inhibitors with enhanced membrane permeability and bioavailability, or optimizing administration routes, is critically important (85, 86). Future advances are expected to resolve these challenges, enabling safer and more effective therapeutic applications of PTP1B inhibitors in atherosclerosis treatment.

## 5 CRISPR-Cas systems as novel therapeutic platforms for atherosclerosis management

The CRISPR/Cas9 (Clustered Regularly Interspaced Short Palindromic Repeats) genome editing platform has revolutionized genetic engineering through its precision in manipulating mammalian genomes. This technology has enabled unprecedented capabilities in functional genomics research and therapeutic development for monogenic disorders, positioning it as a transformative modality in cardiovascular pathobiology (87, 88).

Although CRISPR/Cas9 technology demonstrates significant therapeutic promise for atherosclerosis, associated risks, including off-target effects, toxicity of delivery vectors, and limited editing efficiency, require further investigation (89).

Among various CRISPR-Cas variants, the Type II CRISPR-Cas system from Streptococcus pyogenes (SpCas9) remains the most extensively characterized and widely utilized in biomedical applications due to its high editing efficiency and programmable specificity (87, 90). In a mouse model receiving AAV-CRISPR/Cas9-mediated Ldlr gene correction, partial restoration of Ldlr expression effectively improved the atherosclerotic phenotype, resulting in reduced total cholesterol, low-density lipoprotein cholesterol, and triglyceride levels, diminished macrophage infiltration, and smaller plaques, with no significant off-target effects detected (91).

In the presence of iron, hepatic kupffer cells (KCs) have been shown to mediate critical metabolic processes under iron-replete conditions, particularly through ABCA1-dependent transfer of low-density lipoprotein-derived cholesterol to hepatocytes - a key pathway in systemic lipid homeostasis (92). Complementing these findings, mechanistic studies using the Huh7 human hepatocyte model revealed that MFGE8 overexpression, mediated through its conserved FV/FVIII domains, significantly correlates with enhanced coronary artery disease susceptibility and atherogenesis progression (93).

Of particular clinical relevance, VERVE-101 represents a breakthrough CRISPR-based therapeutic candidate utilizing adenine base-edited mRNA combined with PCSK9-targeting siRNA, delivered via an optimized lipid nanoparticle (LNP) system for single-dose intravenous administration. This innovative approach enables durable modulation of cholesterol metabolism through precision genome editing (57).

Building on these findings, predecessors developed an AAV-compatible dCas9 repressor system (dSaCas9KRAB) demonstrating efficient PCSK9 silencing *in vivo*, establishing proof-of-concept for CRISPR interference strategies in cardiovascular disease management (94). Further validation comes from ANGPTL3 gene silencing experiments in murine models, achieved significant reductions in atherogenic lipid parameters including LDL-C and triglycerides (95, 96). These collective advances underscore the transformative potential of CRISPR/Cas9 systems in developing targeted therapies for atherosclerosis (97).

## 6 MSC-based therapeutic strategies for atherosclerosis management

Inflammation, a fundamental host defense mechanism against pathogenic invasion, plays paradoxical roles in atherosclerosis progression by mediating both protective and pathological responses (88). Atherogenesis is intrinsically regulated through inflammatory cascades that orchestrate immune cell activation, endothelial dysfunction, and metabolic dysregulation across all disease stages (98). Central to this process is endothelial cell (EC) dysfunction, which manifests as impaired nitric oxide (NO) bioavailability due to eNOS (endothelial nitric oxide synthase) uncoupling - a hallmark mechanism linking hemodynamic stress and lipid metabolic disorders to atherosclerotic plaque development (13).

This pathophysiological continuum creates a self-perpetuating cycle where chronic endothelial inflammation promotes plaque vulnerability through matrix metalloproteinase activation and necrotic core expansion. Therapeutic interventions targeting this inflammatory-endothelial axis therefore represent critical strategies for atherosclerotic plaque stabilization.

### 6.1 Therapeutic efficacy of mesenchymal stem cells (MSCs)

Mesenchymal stem cells (MSCs), defined as multipotent stromal cells with tri-lineage differentiation capacity and immunomodulatory properties, have emerged as promising biotherapeutic agents for atherosclerosis intervention (99, 100). Their therapeutic efficacy stems from multimodal mechanisms: (1) Anti-inflammatory reprogramming: MSC-secreted paracrine factors (TSG-6, IL-10, TGF-β) induce macrophage polarization toward the M2 phenotype, attenuating pro-inflammatory cytokine storms in atherosclerotic lesions (101). (2) Plaque microenvironment modulation: Through dynamic crosstalk with plaque-resident cells, MSCs regulate immune cell infiltration profiles, reducing CD68+ macrophage density while increasing regulatory T-cell populations (102–104). (3) Endothelial homeostasis restoration: MSC-derived extracellular vesicles enhance eNOS recoupling via miR-126-3p delivery, counteracting oxidative stress-induced EC apoptosis (105).

These pleiotropic actions collectively stabilize vulnerable plaques by increasing fibrous cap thickness (>65% vs. controls) and reducing lipid core size (41.2% decrease, *p* < 0.01), as demonstrated in recent clinical-phase trials (102–105).

### 6.2 Functional mechanisms of MSCs

Emerging evidence has established an intricate link between small extracellular vesicles (sEVs) and the functional properties of mesenchymal stem cells (MSCs) (105, 106). These nanoscale vesicles, generated through the fusion of multivesicular bodies with the plasma membrane, serve as critical intercellular messengers containing bioactive cargo including mRNAs, microRNAs, proteins, and organelle components. Experimental evidence demonstrates that when MSCs are co-cultured with ox-LDL-stimulated endothelial cells, they significantly upregulate interleukin-8 (IL-8) and macrophage inflammatory protein-2 (MIP-2) expression, subsequently activating the endothelial nitric oxide synthase (eNOS) system. This activation cascade enhances nitric oxide (NO) production and improves endothelial cell functionality (107).

Furthermore, MSC-derived Wnt proteins have been shown to activate the β-catenin-dependent Wnt signaling pathway, effectively mitigating endothelial cell apoptosis through reduction of oxidative stress (108). Notably, mechanistic studies utilizing MSCs derived from patients with atherosclerosis and type 2 diabetes mellitus (T2DM) have provided crucial insights into NF-κB-mediated immunoregulatory pathways (109).

While current research in stem cell therapy continues to reveal complex regulatory networks requiring further investigation, particularly regarding factors modulating stem cell biological functions, MSC-based approaches remain a promising therapeutic strategy for atherosclerosis management.

### 6.3 Key risks and limitations of MSCs

A major meta-analysis on MSC safety, integrating 62 randomized clinical trials (*N* = 3,546 participants), revealed significant risks including transient fever within 48 h post-administration (OR 3.65, 95% CI 2.05–6.49, *p* < 0.01) and increased incidence of administration-site adverse events such as bleeding, swelling, pruritus, pain, or local infection (OR 1.98, 95% CI 1.01–3.87, *p* = 0.05) (110). Furthermore, MSCs' high proliferative capacity and tumor-homing potential enable recruitment into tumor microenvironments in response to hypoxia or pro-inflammatory cytokines (e.g., IL-1β, TNF-α, IFN-*γ*). These tumor-associated MSCs differentiate into cancer-associated fibroblasts (CAFs), which secrete pro-angiogenic and immunosuppressive factors, including PDGF, FGF, VEGF, IL-6, and IL-8, that promote cancer cell survival, angiogenesis, immunosuppression, tumor growth, and metastasis (111). Consequently, comprehensive risk assessment of MSC therapy requires further clinical evaluation.

## 7 Conclusion

As a predominant pathology in cardiovascular and cerebrovascular systems, atherosclerosis manifests through intricate and multifactorial pathogenic mechanisms. With advancing insights into its etiological complexity, contemporary research has yielded novel therapeutic strategies demonstrating superior efficacy to conventional approaches. These innovations encompass three primary domains: (1) optimization of existing treatment protocols, (2) development of next-generation pharmacological agents, and (3) exploration of cutting-edge interventions in gene therapy and regenerative medicine.

The advent of nanotechnology has revolutionized pharmaceutical development through its capacity for site-specific drug delivery, significantly enhancing therapeutic targeting and bioavailability at lesion sites. Concurrently, microRNAs (miRNAs) have emerged as promising therapeutic targets, given their regulatory functions in critical atherogenic processes including lipid homeostasis modulation, immunoinflammatory response coordination, and vascular endothelial remodeling.

This paradigm shift in atherosclerosis management reflects a progressive transition from traditional pharmacological interventions toward an integrated multidisciplinary therapeutic framework. Future clinical approaches will likely emphasize the synergistic integration of diverse treatment modalities, potentially offering optimized therapeutic outcomes through personalized combination therapies.
